# Exploration of Preterm Birth Rates Using the Public Health Exposome Database and Computational Analysis Methods

**DOI:** 10.3390/ijerph111212346

**Published:** 2014-11-28

**Authors:** Anne D. Kershenbaum, Michael A. Langston, Robert S. Levine, Arnold M. Saxton, Tonny J. Oyana, Barbara J. Kilbourne, Gary L. Rogers, Lisaann S. Gittner, Suzanne H. Baktash, Patricia Matthews-Juarez, Paul D. Juarez

**Affiliations:** 1 Department of Public Health, University of Tennessee, Knoxville, TN 37996, USA; 2 Department of Electrical Engineering and Computer Science, University of Tennessee, Knoxville, TN 37996, USA; E-Mails: langston@eecs.utk.edu (M.A.L.); sbaktash@utk.edu (S.H.B.); 3 Department of Family and Community Medicine, Meharry Medical College, Nashville, TN 37208, USA; E-Mails: rlevine@mmc.edu (R.S.L.); bkilbourne@mmc.edu (B.J.K.); 4 Department of Animal Science, Institute of Agriculture, University of Tennessee, Knoxville, TN 37996, USA; E-Mail: asaxton@utk.edu; 5 Research Center on Health Disparities, Equity, and the Exposome, University of Tennessee Health Science Center, Memphis, TN 38163, USA; E-Mails: toyana@uthsc.edu (T.J.O.); pmatthe3@uthsc.edu (P.M.-J.); pjuarez@uthsc.edu (P.D.J.); 6 National Institute for Computational Sciences, Oak Ridge National Laboratory, Oak Ridge, TN 37831, USA; E-Mail: grogers3@utk.edu; 7 Department of Political Sciences, Texas Tech University, Lubbock, TX 79409, USA; E-Mail: lisa.gittner@ttu.edu

**Keywords:** exposome, county rates, data reduction, health disparities, geographical variation, premature birth rates, preterm birth

## Abstract

Recent advances in informatics technology has made it possible to integrate, manipulate, and analyze variables from a wide range of scientific disciplines allowing for the examination of complex social problems such as health disparities. This study used 589 county-level variables to identify and compare geographical variation of high and low preterm birth rates. Data were collected from a number of publically available sources, bringing together natality outcomes with attributes of the natural, built, social, and policy environments. Singleton early premature county birth rate, in counties with population size over 100,000 persons provided the dependent variable. Graph theoretical techniques were used to identify a wide range of predictor variables from various domains, including black proportion, obesity and diabetes, sexually transmitted infection rates, mother’s age, income, marriage rates, pollution and temperature among others. Dense subgraphs (paracliques) representing groups of highly correlated variables were resolved into latent factors, which were then used to build a regression model explaining prematurity (R-squared = 76.7%). Two lists of counties with large positive and large negative residuals, indicating unusual prematurity rates given their circumstances, may serve as a starting point for ways to intervene and reduce health disparities for preterm births.

## 1. Introduction

US infant mortality rates (IMRs) are generally higher than European rates and preterm birth is often identified as one of the main explanations for the high US IMR [1]. Preterm birth rates are higher in non-Hispanic black women in the US than non-Hispanic white and Hispanic women (16.8, 10.5 and 11.7 percent respectively in 2011 for births prior to 37 weeks) [2]. In addition, non-Hispanic black IMR continues to be more than twice that of non-Hispanic white [3], despite recent reductions in both populations [4]. The underlying causes of the higher rate of preterm births in non-Hispanic blacks are not fully understood; the Center for Disease Control (CDC) states, “Preventing preterm birth remains a challenge because the causes of preterm births are numerous, complex, and poorly understood” [5].

Black/white racial disparities in preterm birth rates have been found to be associated with a range of social and economic deprivation factors [6] including poverty, disability and low education levels. Preterm birth rates are high even among highly educated black women [7], however, and have remained higher than the white population over generations [8]. Other risk factors which have been associated with black/white preterm birth rate disparities include exposure to fine particulate matter [9], sexually transmitted infections, nutritional status, access to medical care, stress, and intergenerational effects [10].

Geographical variation in black/white, preterm birth disparities may provide insight into possible preventive interventions to reduce disparities between different communities. Some of the variation is explained by known risk factors, but some of the variation might reflect unidentified differences in modifiable risk factors that have implications for reducing rates. Typically investigators aim to validate a preconceived hypothesis, and data collection is limited to a handful of relevant variables. Prompted by progress in measurement of the effect of environmental exposures on health, however, there has been a call to measure more fully the complex relationships between exogenous and endogenous exposures and their effects on personal health across the lifespan [11] leading to population level disparities at a community level. Juarez *et al*. [12] have structured a longitudinal information system to assess the relationships between health outcomes and social-ecological exposure across the physical, built, social and policy environments (see [13], for a detailed description of the public health exposome conceptual model). The concept of the public health exposome implies measurement of complete exposure pathways ranging from environmental toxins to aggregate-level social-ecological factors on human bio-psycho-social systems, and in its complete form is a daunting task. However, with recent advances in informatics and large and longitudinal, publically available electronic data sets, researchers now have unprecedented access to measures describing the effects of a wide range of environmental and social influences on social problems with complex etiologies such as health disparities. In addition to allowing a more complete measurement of exposures, the public health exposome data repository provides opportunities to use data driven methodologies, allowing the data itself to identify predictors of health outcomes, without the need for preconceived hypotheses. Data driven outcomes can be validated with traditional inferential statistics or used to generate and test new hypotheses.

The aims of this study are to investigate the variation in early preterm birth rates across counties, identify social-ecological and environmental factors which account for this variation, and identify counties with unusually high and low preterm birth rates that can be investigated in greater detail to explain disparate outcomes. Using a county-level dataset with roughly 600 variables, we employed computational analysis in order to group highly correlated variables into dense, noise-resilient clusters [14] known as paracliques [15]. This approach allowed inclusion of a large number of different and highly divergent population level variables, reducing the number of variables under review through graph theoretical techniques, which permitted us to apply traditional and otherwise unscalable statistical analysis techniques.

## 2. Materials and Methods

This study applied a data driven approach, taking the example of preterm birth as the outcome of interest. Graph theory and combinatorial analysis, plus spatial and traditional statistical methods were applied. These allowed analysis of these large data sets to provide insights for improving population health. Aggregate, county-level, population health and environmental measures were employed.

### 2.1. Definitions

County prematurity percentage is calculated as the number of singleton births at gestational ages 24–33 weeks, divided by the number of singleton births of gestational age greater or equal to 24 weeks, in each county. Births weeks 34–36 are also traditionally considered preterm but in this study these births were not included in the numerator to increase the ability to differentiate between normal and abnormal.

### 2.2. Data Sources

County prematurity percentage was derived from the CDC public Wide-ranging ONline Data for Epidemiologic Research (WONDER) internet site [16] which is based on natality file data. The source of the natality files is the birth certificate of all recorded live births. Data were downloaded in two separate time-periods: years 2003–2006 and 2007–2011. An annual average rate for the period 2003–2011 was derived to increase the county-level birth sample and to provide a more stable county value. County numbers of singleton births of gestational age 24–33 (the numerator) and county singleton births of gestational age greater or equal to 24 weeks (the denominator) were downloaded to calculate the county prematurity percentage. Births before gestational age 24 weeks were not included in the numerator or denominator due to concerns over variation in reporting of number of live births at this extremely preterm gestational age. All races were included. Only counties with greater than 100,000 persons are geographically identified in the publically-available CDC data source giving 524 counties that could be linked by county code to other data sources. Counties with less than 100,000 persons were identified by state only, and were not included in this study. 77.4% of all singleton births of gestational age greater or equal to 24 weeks were included in the geographically identified counties.

Data for the county explanatory variables were derived from a number of sources. The US 2004 national natality file (excluding non-singleton births, non-US residents and births before 24 weeks or unknown gestation) provided by the CDC [17] was used to derive county total and race-specific county mean of mother’s age, and proportion of mothers who are married. Similarly, total and race-specific county mean birth weights were calculated using births between 37–41 weeks. The US 2002 national natality file [18] (with the same exclusions as the 2004 file) provided county proportion of mothers who started prenatal care after 3 months or did not receive prenatal care, proportion of mothers who smoke and proportion with number of years of education over 15 years. The 2004 file was selected as the most recent file with county geographical identifiers. Variables that were missing information due to a 2003 revision of the birth certificate were taken from the 2002 file. For both the 2002 and 2004 natality files, county measures were not included when the denominator was less than 30 births. Another 556 explanatory variables were derived from the Meharry public health exposome database, providing county level exposure measures of the natural, built, social and policy environments, including health care provision, among others. Measures of average fine particulate matter air pollution were collected from the CDC Wonder site [19]; data for the prevalence of diabetes, obesity, and diabetes were obtained from CDC Interactive Atlases [20]; data concerning health services and additional socio-economic indicators were from the Area Resource File [21]; county level estimates of black residential isolation were obtained from the publicly available web site of the Arizona State University GeoDa Center [22]; and measures of socio-economic characteristics of a county were collected from the 2000 and 2010 United States Census of Population as compiled by GeoLytics, Inc. (East Brunswick, NJ, USA). GeoLytics produces estimates based on US Census Bureau data and limited population estimates. Additionally, county measures of sexually transmitted infection (STI) levels were obtained from CDC Interactive Atlases [23] and HIV and drug related mortality rates from CDC Wonder [24]. Three variables (two representing black population proportion and one representing number of hot days) were log transformed to correct normality (Shapiro-Wilk’s < 0.8). The prematurity percentage data was linked by county to the Meharry public health exposome database, leaving 520 counties for analysis.

### 2.3. Analysis

A first goal was to assign variables to subsets, so that variables within a subset possess some quantifiable measure of similarity. Foundational to our approach was the use of graph theoretical algorithms and clique-centric tools, which have been shown to outperform conventional clustering methods in numerous applications. See, for example, [14]. It is noteworthy that, unlike with most traditional clustering methods, cliques need not be disjoint. A vertex may lie in more than one clique, just as a variable may be involved in more than one latent factor. Moreover, the usual clustering goal is to maximize edge density. Observe that a cluster’s density is maximized with clique by definition. We recognized, of course, that data is seldom complete or perfect, and so we relaxed clique’s stringency slightly with the use of the paraclique algorithm, first pioneered in [15], to account for noise. For more information on this approach in the context of health disparities, see [25]. Thus, we began by extracting paracliques, then applying factor isolation using the paracliques most highly correlated to the outcome, next performing backward stepwise regression using the extracted factors as independent variables, and finally analyzing residuals. Variables were denoted by nodes, and edges were weighted with Pearson correlation coefficients. Absolute thresholding at 0.61 was performed using spectral methods [26]. Our focus was thereby reduced from the entire parameter space to tightly connected subsets (clusters) of explanatory variables. Paracliques with median correlation to the prematurity outcome variable of at least 0.38 were retained for further processing. Exploratory factor analysis was then applied to each paraclique, iterating until all factor variables had correlation to the factor of at least 0.45. Oblique rotation, allowing correlation between factors (promax), was employed [27]. The number of factors to be retained was guided by the SAS system (proc factor), using the default proportion criteria, where 100% of the common variance is accounted for by the retained factors [28]. Two variables were excluded before factor extraction: one represented black Protestant rates of adherence, which had missing values for counties with low black proportion, and average life expectancy, which would have resulted in a combination of outcome and non-outcome variables. Factors comprised only of outcome variables were not included in further analysis. The extracted factors were entered into a regression model excluding four factors due to missing values in more than 10% of county values; Race/STI, Mother’s Education, Education Black and Income, Married, Age Black (Table 1). We ran Anselin’s Local Moran’s I statistics to determine whether the dependent variable was spatially clustered. Upon confirmation of the existence of spatial clustering in prematurity level, spatial autocorrelation was considered at all steps. Longitude and latitude county values were converted to miles accounting for the sphericity of the earth (cartesian projection). Spatial random effects in multiple linear regressions were accounted for using a spherical spatial model, with the prematurity percentage (logit transformed for normality) as the dependent variable, and the extracted factors as independent variables. The range value for model residuals was estimated using the variogram model, but we let the mixed model fit the sill and nugget values. The independent variables in the model were eliminated using backward sequential selection based on *p*-values. Using maximum likelihood as the criterion of fit [29] an R-squared value was calculated.

**Table 1:** Paracliques with median pairwise correlation > 0.38 to prematurity and factors extracted from these paracliques (two paracliques made up of outcome variables alone are not shown).

Paraclique	Variables	Extracted Factors	Pearson Correlation of Factor to Logit Prematurity	N
1.	Log (percent black/African Am pop, 2000)	Black population proportion	0.686	520
	Black isolation index, 2000			
	Log (% Black, 2008)			
	Rate gonorrhea, 2011	STI	0.710	520
	Rate chlamydia, 2011			
	Black Protestant-rates of adherence per 1,000 population, (2010)	Excluded (missing values)		
	Low birth weight (<2500 gram)	Excluded (outcome variables)		
	Very low birth weight (<1500 gram)			
	Premature birth; singleton births 24–33 weeks/singleton births ≥ 24 weeks			
2.	Births to unmarried women	Married mother	−0.749	520
	% married mothers			
	Percent Medicaid eligible female, 2004	Medicaid	0.428	520
	Medicaid eligible total, 2004			
	Percent Medicaid eligible male, 2004	Medicaid males	0.465	520
	Percent food stamp/SNAP * recipients, 2005	Poverty and teen birth	0.671	520
	Poverty rate, 2008			
	Child poverty rate, 2008			
	Median household income			
	Births to women under 18			
	SNAP *authorized stores/ 1000 pop, 2008	Not included (below threshold for factors)		
	Free lunch %, 2008			
3.	Percent white population, 2000	Race/STI	0.468	439
	Rate HIV mortality			
	Rate HIV prevalence			
	Rate syphilis			
4.	Number of days with maximum temperatures ≥ 90 degrees Farenheit	Temperature/Divorce	0.433	512
	Daily maximum temperature 1999–2009			
	Divorced rate			
	Average minimum daily temperature	Temperature/Land	0.441	520
	Average nightly land surface temperature			
	Average daily land surface temperature	Sunlight	0.219	520
	Average direct solar radiation in kilojoules per square meter			
	Percent less than 65 no health insurance	No Health Insurance	0.203	520
	Percent females less than 65 no health insurance			
5.	Adult diabetes rate	Diabetes/Obesity	0.603	520
	Age-adjusted rates of leisure-time physical inactivity, 2009			
	Adult obesity rate			
	Age adjusted obesity rates, 2009			
	Average life expectancy	Excluded (outcome)		
6.	Rate of hospital admissions, 2005	Hospital Admissions	0.465	520
	Rate of short term general hospital admissions, 2005			
	Rate of short term community hospital admissions, 2005			
	Rate of medical/surgical intensive care beds, 2006	Hospital Beds1	0.427	520
	Rate of operating rooms, 2005			
	Rate of licensed beds short term hospital, 2005			
	Rate of licensed beds total hospital, 2005			
	Rate medical/surgical adult beds			
	Rate of hospital beds, 2005	Hospital Beds2	0.467	520
	Rate of short term general hospital beds, 2005			
	Rate of total inpatient beds			
	Rate surgical operations inpatient	Surgical Operations	0.357	520
	Rate surgical operations total			
	Rate hospital beds	Hospital Beds3	0.416	520
	Rate of short term community hospital. beds, 2005	Not included (below threshold for factors)		
7.	Average daily maximum heat index	Heat Index	0.492	511
	Daily maximum heat index 1999–2009			
	Log (number of days with maximum temperatures ≥ 100 degrees Farenheit)			
	Index combining average fine particulate matter with average daily maximum temperature.	Pollution	0.528	512
	Normalized average fine particulate matter (2003–2008) plus average daily maximum heat index 1999–2009			
8.	Per capita income, 2005	Income/Private Practice	−0.360	520
	Rate dentists private practice, 2007			
	Births to women over 40	Mother’s Age	−0.563	520
	Mean mother age			
	Percent mother’s education > 15 years	Mother’s Education	−0.529	453
9.	Median household income, white	Medicare Disabled/Income	0.411	520
	Percent Medicare enrollment disabled hospital insurance, 2005			
	Percent Medicare enrollment disabled, 2005			
	Percent Medicare enrollment disabled supplementary medical insurance, 2005			
	Less than high school white female %, 2010	Low Education White	0.451	520
	White low education %, 2000			
10.	College black female %, 2010	Education Black	−0.491	387
	College black male %, 2010h			
	Black high education %, 2000			
	Percent mothers education > 15 years, black			
	Mean mother age, black	Income, Married, Age Black	−0.563	443
	Median household income, 2000, black			
	Per capita income 2010, black			
	Married mothers, black			
11.	GINI inequality index, 2000	Income Inequality	0.431	520
	Theil inequality index, 1990			
	Gini index, 2010			
12.	Black low education %, 2000	Low Education, Black1	0.410	520
	Black male low education %, 2000			
	Black female low education %, 2000	Low Education, Female Black2	0.471	520
	Less than high school black male %, 2010	Not included (below threshold for factors)		
13.	Median age black/African American female, 2000	Aged Black	0.443	520
	Percent African American females 65+, 2000			
	Percent African American males 65+, 2000			
14.	Separated/widow/divorced white %, 2010	Separated White1	0.404	520
	Separated/widow/divorced white female %, 2010			
	Separated/widow/divorced white male%, 2010	Separated White2	0.312	520
	Percent divorced females			
15.	Teen birth rate	Religion/Teenbirth/Stores	0.510	520
	Convenience stores with gas/ 1000 pop, 2008			
	Evangelical Protestant rates of adherence per 1,000 population, (2010)			

* SNAP Supplemental Nutrition Assistance Program.

The county studentized residuals from the final model were mapped in 5 groups to examine the geographical distribution of the outliers; <−2.0, −2 to −1.5, −1.5 to 1.5, 1.5 to 2.0 and >2.0. Those counties with studentized residuals <−1.5 were classed as an overpredicted group, while those with studentized residuals >1.5 were classed as an underpredicted group, and those between −1.5 to 1.5 formed an intermediate group (residual groups). As regression modelling does not guarantee that the overpredicted and underpredicted counties are equivalent in terms of the explanatory variables, these groups were compared by key variables such as county poverty prevalence, percent African-American and proportion not starting prenatal care in the first three months of pregnancy using the Kruskal-Wallis test. Premature birth rate was also compared between the three groups. In a sensitivity analysis to examine the effect of including a variable representing prenatal care in the regression, (prenatal care had not been included because the correlation of the paraclique representing prenatal care to the outcome was less than the threshold for factor extraction), backward selection starting with the same factors entered into the original regression plus a variable representing prenatal care, was carried out. The regression model was partially reduced to a point retaining the variable representing prenatal care, and the residuals used to make the overpredicted, underpredicted and intermediate groups. The groups were compared by the prenatal care variable.

Level of significance for statistical tests was set at *p* < 0.05. Statistical analyses were performed in SAS version 9.3 (SAS Institute, Cary, North Carolina, USA), and mapping in ESRI’s ArcGIS Desktop 10.2 (ESRI Inc., Redlands, CA, USA).

## 3. Results

**Figure 1:**
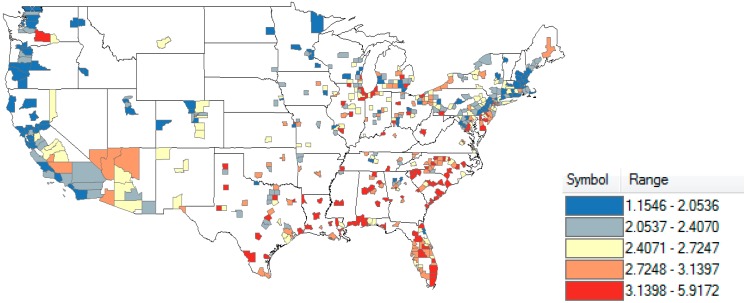
County prematurity percentage. N = 520.

County prematurity percentages ranged from 1.155/100 in Marin County, California to 5.917/100 in Hinds County, Mississippi. A high degree of correlation between the two periods (years 2003–2006 and 2007–2011) was found; R = 0.905, *p* < 0.0001 (Pearson’s correlation). There was therefore a tendency for counties with higher percentages in the first period also to be higher in the second period, indicating a real rather than a random finding. Geographically, higher county prematurity percentages were more commonly found in the southeastern United States, with lower values in the northeastern states and in the West (Figure 1).

Forty-eight paracliques were generated using 284 county-level variables. Paraclique sizes ranged from 3 to 34 variables. Seventeen paracliques had an absolute median correlation to county preterm birth rate at or above 0.38. 33 factors were extracted from these paracliques (Table 1). These factors covered a wide range of constructs, including black proportion, obesity and diabetes, STI rates, mother’s age, income, marriage rates, pollution and temperature among others. Some factors comprised variables from different concepts (e.g., poverty and teen-birth), while others combined different variables within a single concept.

**Figure 2:**
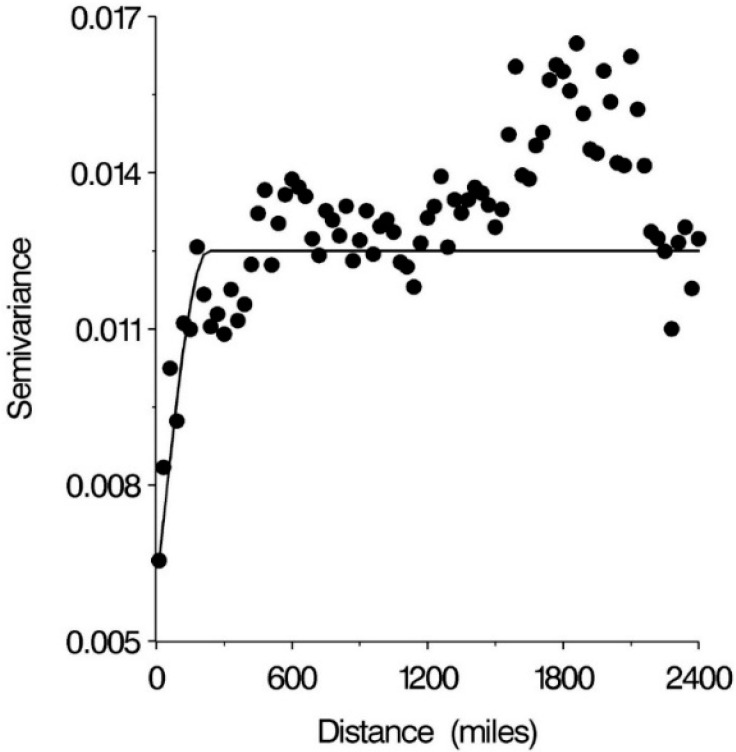
Spatial variogram used to determine range, scale and nugget used in spherical covariance matrix. The parameters used in the model and as shown in the solid line on the graph were nugget 0.006, range 230 miles and scale 0.0065.

A variogram of the residuals showed an increase in variance between county pairs to a range of about 230 miles (Figure 2). Correction for spatial autocorrelation with a spherical covariance matrix improved fit of the model (as measured by the AIC Akaike Information Criterion) from −709.6 to −811.1. The regression model was reduced by backward selection to leave nine independent variables (nine of the extracted factors as detailed in Table 1); black proportion, STI, married mother, diabetes/obesity, medicare disabled/income, no health insurance, pollution, mother’s age and income/private practice, each with a statistically significant effect on the outcome. Variables married mother and mother’s age were negatively associated with logit county prematurity percentage, while the other variables were positively associated (Table 2).

**Table 2:** Final regression model of outcome logit county prematurity percentage and extracted factors as independent variables using a spherical covariance matrix (N = 512 counties).

Factor	Parameter Estimate	Standard Error	*p*	AIC
STI	0.04431	0.00921	<0.0001	−837.6
Black proportion	0.05950	0.01000	<0.0001	
Married Mother	−0.07493	0.01001	<0.0001	
Diabetes/Obesity	0.02879	0.01098	0.0090	
Medicare Disabled/Income	0.02275	0.00820	0.0058	
Pollution	0.03426	0.01095	0.0020	
Income/Private Practice	0.02481	0.00951	0.0094	
Mother’s Age	−0.04749	0.01324	0.0004	
No Health Insurance	0.03449	0.00799	<0.0001	

**Figure 3:**
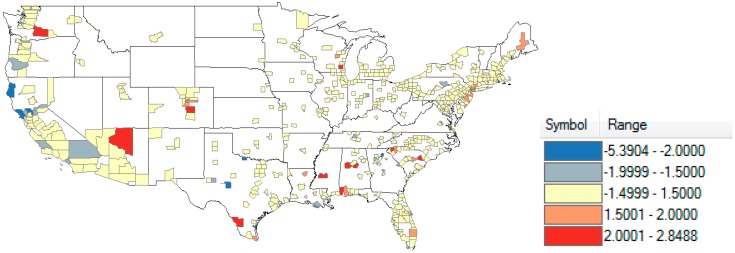
Mapping of residuals from reduced model taking into account spatial autocorrelation N = 512.

The map of the residuals from the reduced model using a spherical covariance matrix (Figure 3) shows a similar geographical distribution to that of county prematurity percentage itself, with lower residuals in the West.

**Figure 4:**
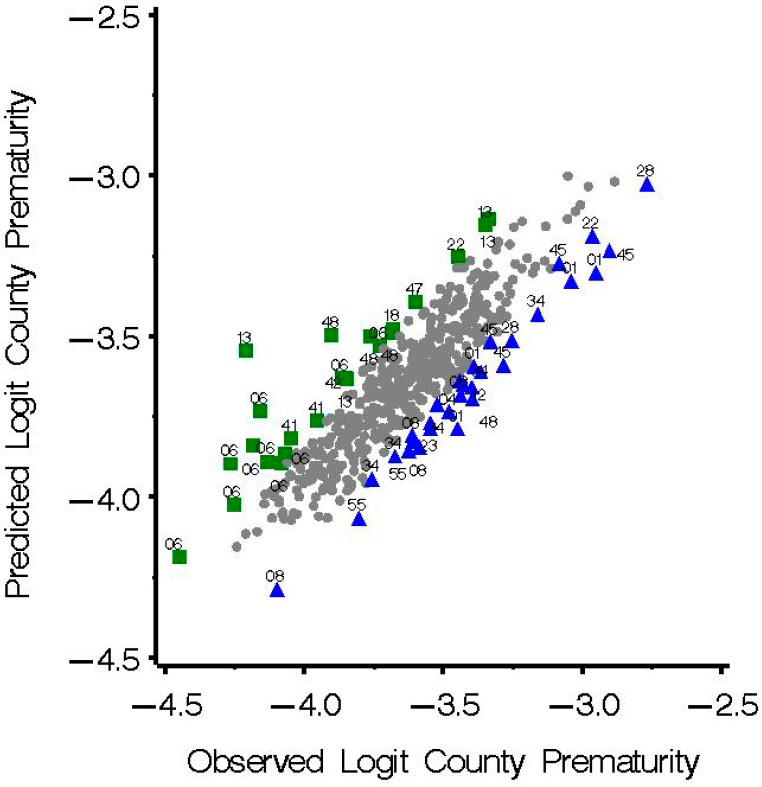
Observed logit of county prematurity percentage *versus* predicted (N = 512) in the overpredicted group (studentized residuals <−1.5), the underpredicted group (studentized residuals >1.5) and the intermediate group (studentized residuals −1.5 to 1.5).

The graph of the observed outcome, logit of county prematurity percentage, *versus* expected (Figure 4) shows that the counties in the underpredicted and overpredicted groups were distributed throughout the range of prematurity percentages. County prematurity percentage was significantly lower in the overpredicted than in the underpredicted group (*p* < 0.0001). In comparing key county variables (Table 3), significant differences between the residual groups in most variables examined were not found. Median proportion non-Hispanic white population was higher in the intermediate group than in the over and the underpredicted groups (*p* = 0.0079). Median proportion non-Hispanic African-American population was higher in the underpredicted *versus* overpredicted counties but this difference was not statistically significant. Variables representing prenatal care not received in first trimester and mother reporting smoking were found to differ significantly between the three groups. When the prenatal care variable was included in the regression model the difference between the groups in prenatal care (proportion of mothers not receiving care in first trimester) remained significant.

**Table:** 

Counties where studentized residuals < − 2.0	Counties where studentized residuals > 2.0
Hall County, Georgia	Mobile County, Alabama
Humboldt County, California	Shelby County, Alabama
Wichita County, Texas	Florence County, South Carolina
Sonoma County, California	Webb County, Texas
Yolo County, California	Pickens County, South Carolina
Marin County, California	Tuscaloosa County, Alabama
Tom Green County, Texas	Essex County, New Jersey
	El Paso County, Colorado
	Yakima County, Washington
	Rankin County, Mississippi
	Waukesha County, Wisconsin
	Hinds County, Mississippi
	Coconino County, Arizona

**Table 3:** Median values of selected predictor variables in county groups divided by studentized residuals.

	Overpredicted Group, Studentized Residuals <−1.5	Studentized Residuals −1.5–+1.5	Underpredicted Group, Studentized Residuals > +1.5	*p*
Prenatal care not received in first 3 months of pregnancy	16.3%, N = 19	14.8%, N = 405	18.5%, N = 23	0.0197
Poverty rate	14.1%, N = 23	12.2%, N = 459	12.8%, N = 30	0.2901
Non-Hispanic Black Proportion	5.5%, N = 23	7.6%, N = 459	9.6%, N = 30	0.3929
Non-Hispanic White Proportion	64.4%, N = 23	77.4%, N = 459	64.7%, N = 30	0.0079
Age Adjusted Obesity 2009	27.8%, N = 23	28.4%, N = 459	27.2%, N = 30	0.2802
Rate Gonorrhea	116.4/100,000, N = 23	67.8/100,000, N = 459	62.3/100,000, N = 30	0.9458
Mean Mother Age	27.0, N = 23	27.2, N = 459	27.1, N = 30	0.6735
Mother smoker	10.7%, N = 9	12.9%, N = 383	8.6%, N = 23	0.0065
Married mothers	61.2%, N = 23	64.2%, N = 459	65.8%, N = 30	0.6742
Mothers education >15 years	26.3%, N = 19	25.6%, N = 405	27.1%, N = 23	0.7891

## 4. Discussion

A main aim of our work has been to apply state-of-the-art computational tools to generate hypotheses that can explain variation in health outcomes. A major focus has been not only to suggest what might be useful to study, but also to identify spatial units that might be a good place to look. Future investigations may attempt to explain why certain counties have a lower prematurity percentage than expected from the predictors included in the model (resilient counties), potentially revealing protective mechanisms. A comparison of counties that are similar in expected rates but quite different in observed could allow previously unidentified mechanisms to become more apparent. Although some of the counties may be outlying by chance, the concentration of positive or negative residual counties in the same states points against the role of chance. We found a concentration of overpredicted counties in California. Detailed follow-up studies to investigate the mechanisms underlying the resiliency *versus* vulnerability of extreme counties are required. Of course the role of reporting differences or reporting errors needs to be eliminated as a possible reason for differences.

In a previous study investigating geographical variation in black infant mortality rate, counties with rates significantly less than that predicted by the model were identified as resilient counties [30]. In these counties the racial disparities in infant mortality were eliminated despite a lower educational attainment and higher levels of poverty in blacks compared to whites in the same counties. It was suggested that these counties could provide models for success in elimination of health disparities independent of socioeconomic status. In a study examining disparity in HIV mortality before and after the introduction of highly active antiretroviral treatment (HARRT) [31], the effect of place was found to be important, with some communities, particularly those with high pre-HAART disparities, more vulnerable than others. It was found that some of the counties with particularly high disparities were contiguous, suggesting a shared experience.

Our methodology allowed a relatively hypothesis-free approach to the investigation of county variation in prematurity rates. The methodology was not completely hypothesis free, because prior assumptions still influenced the choice of variables that were included in the data set, but a wide variety of variables was provided. An analysis strategy capable of handling roughly 600 explanatory variables was needed. Two strategies were used to reduce the number of independent variables to a manageable level for use in regression. First, scalable graph algorithms were employed to produce strongly correlated sets of variables (paracliques). Second, filtering by strength of correlation of the paraclique to the outcome, with subsequent extraction of the underlying constructs contained in the selected paracliques, was performed. Traditionally, exploratory factor analysis has been used to reveal the underlying structure of a set of interrelated variables from a questionnaire without imposing any preconceived structure on the outcome, identifying the number of constructs and underlying factor structure of a group of correlated variables [32]. We have used the same principles, using data from population data sets rather than questionnaires to extract common factors. These factors can then be input as dependent variables into regression analysis. Using these methods we managed to include many more variables than are usually included, using the range of data that is now available.

The predictors themselves may provide directions for future investigation. These predictors were extracted from an extensive database, allowing a data-driven approach to identify them. This provided a higher likelihood of finding relevant and previously unidentified factors than if a limited number of variables had been pre-selected based on previous research. The predictors found to be significant in the regression model (county black proportion, STI rates, diabetes/obesity rates, Medicare disabled enrollment/income rate, percentage of married mothers, pollution, mother’s age, no health insurance rates) should reflect factors that are common enough and increase risk enough to show an effect at the aggregate level.

There has been recent interest in maternal obesity as a predictor of preterm birth. In a large Swedish cohort [33], the risk of both medically indicated and spontaneous preterm birth were shown to increase with body mass index and obesity. The high rate of obesity in the US, about twice that of Sweden, was noted to have extensive implications for preterm birth rate. The effect of obesity without glucose dysfunction, however, was not examined in this study. We found that obesity, diabetes and physical activity were highly correlated, as expected, since obesity is an important precursor of diabetes. Diabetes has been associated with preeclampsia which can lead to a medically indicated preterm birth [34]; additionally there is evidence for an association of diabetes with spontaneous preterm birth. In a US cohort study, both gestational diabetes and glucose dysfunction not severe enough to be termed gestational diabetes were shown to increase the risk of spontaneous preterm birth [35].

The association we found of Medicare-disabled-enrolled with preterm birth is probably a reflection of the general population of the county rather than a reflection of the population of mothers. Medicare assistance is available to certain qualifying patients under the age of 65 with long-term disabilities such as end-stage renal disease. This implies that the counties with the highest burden of chronic disease are the same counties as those with the highest preterm birth rates, suggesting an underlying common mechanism such as poverty-related factors leading to both high rates of Medicare-disabled-enrolled and high rates of preterm births in the same counties.

The validity of the STI data is a potential limitation of this study. County STI rates are based on surveillance data using reported cases to the Centers for Disease Control and Prevention (CDC) [23]. Reporting from public sources is thought to be more complete than reporting from private sources [36], and a reporting bias is possible with increased reporting in lower socioeconomic groups. Nevertheless, previous studies have shown an association between STI and preterm birth [37]. In a study based on a cohort of low income women in South Carolina, women diagnosed with trichomoniasis, gonorrhea, or chlamydia/non-gonococcal urethritis had increased risk of very preterm and late preterm birth. In a multivariate model, STI was more strongly associated with very preterm than with late preterm birth [38]. It is possible that gonorrhea and chlamydia county rates as included in the STI factor in our study are markers for an associated risk factor for preterm birth such as bacterial vaginosis [39].

Other limitations of this analysis include the cross-sectional and aggregate (county-level rather than individual level) nature of the data. In addition, the analysis indicates predictors of the county preterm birth rate rather than causal pathway. Another limitation relates to the data including problems such as missing values, variables that are distributed non-normally and a combination of outcome and non-outcome variables in the same data-set. Additionally, results are not provided for population groups at different risks of preterm birth. Risk factors might differ between different races and ethnicities and for different preterm birth types.

## 5. Conclusions

In summary, this study provides a county level analysis of social and environmental predictors of preterm birth, in US counties of greater than 100,000 persons, using a very large data set of routinely collected variables. A novel methodology for analysis of a very large data set was used. Avenues for future investigation into the common and most operative forces leading to preterm birth were suggested, as well as identification of resilient counties for investigation of potential protective mechanisms. Although preterm birth rates were available by race, many of the explanatory variables were not, so this analysis was performed at the total county population level. While most of the predictors of premature birth have been previously identified in individual studies, no single study has identified all of them collectively. In this manner, this exemplar validates the application of a data-driven approach and computational methods to the large, combined, disparate data sets encompassed by the public health exposome and the examination of complex social problems such as health disparities. Other data-driven analytical approaches in a more network directed analysis have shown promise with this data [25] and will be explored further. In future studies, we intend to replicate these methods with other health disparities that have been found to have complex etiologies, such as lung cancer, breast cancer, cardio-vascular disease, and homicide. In addition, in the next phase of our research, we are working on growing the public health exposome database from 600 to over 15,000 variables. The public health exposome provides a promising approach for identifying complex underlying factors associated with health disparities, identifying communities at high and low risk, and developing appropriate targeted interventions.
